# Permeability of blood-tear barrier to fluorescein and albumin after application of platelet-activating factor to the eye of the guinea pig

**DOI:** 10.1080/09629359791532

**Published:** 1997-12

**Authors:** J. L. Van Delft, F. Meijer, J. A. Van Best, N. J. Van Haeringen

**Affiliations:** 1 Department of Ophthalmology Leiden University Medical Center Albinusdreef 2 Leiden 2333 ZA The Netherlands; 2 The Netherlands Ophthalmic Research Institute Amsterdam The Netherlands

## Abstract

One of the inflammatory responses of the eye to local application of platelet-activating factor (PAF) is oedema of the conjunctiva, caused by extravasation of plasma. Aim of the study was to investigate if fluorescein would leak from the blood into the tears together with plasma protein after application of PAF to the eye. Fluorescein was given intraperitoneally 30 min prior to application of 25 μl of 0.1% solution of PAF. Thirty min after PAF the tear film was collected by washing the surface of the eye with 25 μl of phosphate buffered saline (PBS). Fluorescein in eye washings and in plasma was measured by fluorophotometry and albumin by immunodiffusion. Both fluorescein and albumin appeared in a related fashion in tears, being absent in washings of placebo-treated control eyes. Extravasation of fluorescein can be used as a measure for plasma leakage in the conjunctiva with the advantage over the Evans Blue method that the former is a non-invasive method.


Mediators of Inflammation, 6, 381–383 (1997)

### Permeability of blood–tear barrier to fluorescein and albumin after application of platelet-activating factor to the eye of the guinea pig

J. L. van Delft[1,CA], F. Meijer[2], J. A. van Best[1] and N. J. van Haeringen[2]

[1]Department of Ophthalmology, Leiden University Medical Center, Albinusdreef 2, 2333 ZA Leiden; and [2]The Netherlands Ophthalmic Research Institute, Amsterdam, The Netherlands

[CA]Corresponding Author
Tel: (+31) 71 526 1776/2378
Fax: (+31) 71 524 8222

**One of the inflammatory responses of the eye to local application of platelet-activating factor (PAF) is oedema of the conjunctiva, caused by extravasation of plasma. Aim of the study was to investigate if fluorescein would leak from the blood into the tears together with plasma protein after application of PAF to the eye. Fluorescein was given intraperitoneally 30 min prior to application of 25 µl of 0.1% solution of PAF. Thirty min after PAF the tear film was collected by washing the surface of the eye with 25 µl of phosphate buffered saline (PBS). Fluorescein in eye washings and in plasma was measured by fluorophotometry and albumin by immunodiffusion. Both fluorescein and albumin appeared in a related fashion in tears, being absent in washings of placebo-treated control eyes. Extravasation of fluorescein can be used as a measure for plasma leakage in the conjunctiva with the advantage over the Evans Blue method that the former is a non-invasive method.**

**Key words:** Platelet-activating factor (PAF), Fluorescein, Albumin, Guinea pig, Blood–tear barrier

### Introduction

The blood–tear barrier, causing the large concentration differences between tear and blood components, is formed by the acinar and ductal cells of the lacrimal gland and by the conjunctival and corneal epithelium at the surface of the eye.

Breakdown of the blood–tear barrier occurs in allergic conjunctivitis, where increased vascular permeability has been described as an important feature.[1] Besides histamine and prostaglandins, platelet activating factor (PAF) takes part in the inflammatory process.[2] On a molar basis PAF appeared to be $10^3$ to $10^4$ times more potent in inducing vascular permeability changes in skin compared with histamine.[3] In guinea pigs topical application of PAF on the eye produces within 30 min a dose-dependent increase of the vascular permeability of the conjunctiva, as determined by measurement of the serum albumin concentration in lavage fluid of the surface of the eye.[1]

The aim of the present study is to investigate if application of PAF to the eye might also cause leakage of intraperitoneal injected fluorescein from blood into the tears together with serum albumin. Both fluorescein and albumin appeared significantly correlated in the eye washings after application of PAF.

### Materials and Methods

Eight female Hartley strain guinea pigs (weight range 350–450 g) were intraperitoneally injected with 0.25 ml/kg body weight of a 20% solution of fluorescein sodium and 30 min later 25 µl of 0.1% solution of PAF in phosphate buffered saline (PBS) was applied to the right eyes. The left eyes served as controls and received 25 µl of PBS.

Thirty min after application of PAF or PBS tears were collected by washing the surface of the eye with 25 µl of PBS, using a polypropylene micropipette, avoiding touching the eye. After three forced blinks the lavage fluid was collected and stored at −20°C until use. Blood was collected by heart puncture of the animals, anticoagulated with heparin and centrifuged for separation of the plasma.

Albumin concentration in lavage fluid was determined using a radial immunodiffusion method in agar plates (1.5%), containing guinea pig albumin antiserum in 1:100 dilution. Various concentrations of guinea pig albumin were used as standard. Samples (5 µl) were tested in appropriate dilutions.

Fluorescein was measured in lavage fluid and plasma by fluorophotometry, using a fluorophotometer (Fluorotron Master, Ocumetrics Inc., Mountain View, CA, USA). For measure-

© 1997 Rapid Science Publishers ment in lavage fluid 20 μl was diluted in 0.5 ml of PBS in a 5 mm-cuvette and plasma 20 μl was diluted in 4 ml of PBS in a 10 mm-cuvette. Fluorescence was measured in the range 510–600 nm, using an excitation wavelength of 430–490 nm.

PAF was purchased from Cayman (Ann Arbor, MI, USA), guinea-pig albumin antiserum and guinea-pig albumin from Nordic (Tilburg, The Netherlands). Animals were housed and cared in accordance with rules and regulations of the European Community Board concerning the use of experimental animals (86/609/CEE).

### Results

Fig. 1 shows a significant correlation of the fluorescein concentration, expressed in percentage of the plasma concentration, and the albumin concentration in the lavage fluid of eyes, treated with PAF, in eight animals. The concentrations of fluorescein and albumin in lavage fluid of PBS treated eyes were below the detection limit of respectively 3 ng/ml and 15 μg/ml. The measured concentrations of fluorescein in plasma varied from 11.2 to 25.0 μg/ml (mean $16{\cdot}3 \pm 1{\cdot}7$) and in lavage fluid from 3 to 257 ng/ml (mean $147{\cdot}5 \pm 38$). Albumin concentrations varied from 0 to 376 μg/ml (mean $188 \pm 48$).

### Discussion

The variation of the appearance of albumin in the lavage fluid of the eyes in response to local application of PAF is in correspondence with our observations, described earlier.[1] The great variation in response may be explained by variation in the penetration of the outer eye. The resorption depends on the ocular contact time of the eye drop, containing PAF, and on enzymatic hydrolysis of PAF to lyso-PAF by an acetylhydrolase, present in ocular tissue.[4] Several ocular tissues themselves, subsequent to inflammation, are able to produce PAF in ng quantities.[5]

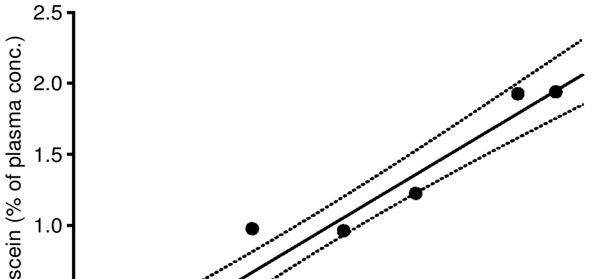

FIG. 1. Relationship between albumin and fluorescein in lavage fluid of eyes of guinea pigs, collected 30 min after treatment with 25 μl of 0.1% PAF. Correlation coefficient 0.98 ($P < 0.0001$, $n = 8$).

Albumin is not produced by the lacrimal gland of the guinea pig[6] and the appearance of albumin on the ocular surface is a result of the flow of plasma protein containing tissue fluid across the epithelium to the ocular surface under the influence of PAF. The lavage fluid from the surface of the eye at this stage contains a mixture of leaking plasma and lacrimal gland fluid from the lacrimal gland.

The amount of fluorescein leaking out of the blood vessels is dependent on the rapidly changing concentration of fluorescein in the blood. Therefore fluorescein concentrations are calculated as percentage of the plasma concentration. The same should apply for serum albumin, however the plasma concentration of about 45 g/l is not likely to change in short-time experiments as carried out in this study. The greater part, more than 90% of fluorescein in blood is bound to protein and this explains the relationship in lavage fluid found for albumin and fluorescein. The determination of albumin in lavage fluid as a measure for conjunctival inflammation is a relatively simple method.

The Evans Blue method for measuring extravasation of plasma in inflamed conjunctival tissue is based on the same principle of comigration of dye with plasma proteins. However it is an invasive procedure where the animals have to be killed for isolation of the conjunctival tissue and extraction of extravasated dye. The concentration of Evans Blue in the tears is too low to be detectable by colorimetry compared with fluorescein, which can be measured fluorimetrically with a detection limit of 1 ng/ml. Measurement of fluorescein in lavage fluid of the eyes is an alternative for Evans Blue with the advantage that it is not necessary to kill the animals.

### References

1. Meijer F, van Delft JL, van Haeringen NJ. Platelet-activating factor: an inflammatory mediator in the acute phase of allergic conjunctivitis in a guinea-pig model. *Mediators Inflam* 1995; **4**: 191–195.
2. Abelson MB, Schaefer K. Conjunctivitis of allergic origin: immunologic mechanism and current approaches to therapy. *Surv Opthalmol* 1993; **38**: 115–132.
3. Hwang SB, Li CL, Lam MH, Shen TY. Characterization of cutaneous vascular permeability induced by platelet-activating factor in guinea pigs and rats and its inhibition by a platelet-activating factor antagonist. *Lab Invest* 1985; **52**: 617–630.

4. Bazan HEP, Tao Y, Hurst JS. Platelet-activating factor antagonists and ocular inflammation. *J Ocul Pharmacol* 1994; **10**: 319–327.
5. Rosenbaum JT, Boney RS, Samples JR, Valone FH. Synthesis of platelet activating factor by ocular tissue from inflamed eyes. *Arch Ophthalmol* 1991; **109**: 410–413.
6. Thörig L, van Agtmaal EJ, Glasius E, Tan KL, van Haeringen NJ. Comparison of tears and lacrimal gland fluid in the rabbit and guinea pig. *Curr Eye Res* 1985; **4**: 913–920.

**Received 15 May 1997;
accepted in revised form 12 June 1997**
